# Designing, Developing, and Evaluating a Stakeholder-Informed Mobile App to Promote Physical Activity in Children

**DOI:** 10.3390/ijerph22091460

**Published:** 2025-09-20

**Authors:** Olga Papale, Emanuel Festino, Lamprini Papargyri, Cristina Cortis, Andrea Fusco

**Affiliations:** 1 Department of Human Sciences, Society and Heath, University of Cassino and Lazio Meridionale, 03042 Cassino, Italy; olga.papale@unicas.it (O.P.); emanuel.festino@unicas.it (E.F.); 2 European University of Technology EUt+, 03043 Cassino, Italy; 3 MindSpin, Nicosia 1678, Cyprus; papargyri1@gmail.com; 4 Department of Medicine and Aging Sciences, University “G. d’Annunzio” of Chieti-Pescara, 66100 Chieti, Italy; andrea.fusco@unich.it

**Keywords:** sedentary behaviors, health promotion, group concept mapping, walking

## Abstract

**Background**: Prolonged sedentary behavior and associated obesity are recognized risk factors for poor health across the lifespan. Globally, data show that many children and adolescents aged 5 to 17 significantly increased their sedentary behaviors during the COVID-19 pandemic, failing to meet recommended physical activity levels and reporting increased smartphone use. While mobile devices and video games have been traditionally linked to physical inactivity, formats like exergaming, which combine gameplay with gross motor activity, offer potential to promote physical activity. However, many digital health tools for children are developed without incorporating feedback from key stakeholders and end-users (e.g., children, teachers, and guardians). Therefore, this paper, within the Walk around the Earth (E-Walk) project, describes a prospective study that aims (1) to identify the most influential factors or characteristics affecting engagement with and usability of a mobile application promoting physical activity among primary school students; (2) to develop a mobile application for children based on the identified factors and characteristics. **Methods**: This project will use a group concept mapping approach to identify the most influential features/factors/characteristics affecting engagement with and usability of an app. By involving primary stakeholders (e.g., children, teachers, guardians, and physical activity experts), the project seeks to align the app’s features with primary end-user needs and motivations. Following the app’s development, its effectiveness in increasing physical activity levels and reducing sedentary behaviors will be evaluated through a mixed-method design, incorporating anthropometric data, validated physical activity questionnaires (Physical Activity Questionnaire for Older Children (PAQ-C) and International Physical Activity Questionnaire (IPAQ)), and engagement metrics. **Conclusions**: The E-Walk project integrates participatory design with educational content and activity-based challenges, representing a multidimensional strategy for promoting health and learning in primary school students. Ultimately, this study contributes to the development of user-informed digital interventions that support sustainable behavioral changes, in line with broader goals of child well-being and digital health promotion.

## 1. Introduction

The prevention of non-communicable diseases, such as obesity and cardiovascular and metabolic conditions, particularly among children, is a major public health goal worldwide [1]. Global data show that many children and adolescents aged 5 to 17 years do not meet the recommended daily levels of physical activity, increasing their risk of overweight and obesity [2,3,4]. In 2022, more than 390 million children and adolescents were classified as overweight, with approximately 8% living with obesity [5], making childhood obesity one of the most serious public health challenges of the 21st century. The Organization for Economic Co-operation and Development/European Union document [6] confirmed the widespread prevalence of inadequate daily levels of exercise among children and adolescents, with important implications for national health systems. The COVID-19 pandemic further aggravated these trends, as digital home-based training was effective for adults [7], whereas children showed increased sedentary behaviors and screen exposure during lockdowns [8]. Several studies [9,10] reported a strong association between excessive screen time, particularly smartphone and tablet use, and sedentary lifestyles. Currently, 74% of European children aged between 7 and 12 years, and 83% of adolescents aged between 13 and 15 years report regular smartphone use, highlighting the widespread integration of digital devices into daily life [11].

Smartphones and tablets, including the software applications that run on these devices, have become an integral part of children’s lives, with substantial increases in usage in recent years [11], and it is unclear whether the (ab)use of such devices can actually be considered as part of “addictive” behavior [12]. While media such as video games have traditionally been associated with inactivity, new forms that combine play with motor activity (active video games or exergaming) may support children in meeting physical activity recommendations [13]. In parallel, a variety of mobile health and fitness apps have emerged, providing behavioral interventions to large population groups. Despite their potential, a critical limitation persists. Many strategies are developed without the involvement of thorough stakeholder feedback. In particular, the perspectives of children, who are the primary end-users, and teachers, who implement these programs within constrained classroom contexts, are often not adequately considered. This lack of participatory design reduces effectiveness, sustainability, and applicability of such interventions. Without a clear understanding of teachers’ challenges and children’s preferences and motivations, many initiatives risk being poorly aligned with their target users, limiting their impact on sedentary behavior reduction.

Nevertheless, previous digital interventions have yielded mixed results. While some school-based programs were able to increase physical activity in the short term, their effects often diminished over time without sustained engagement or contextual support [14]. Moreover, tools developed without active user involvement have frequently reported poor usability and limited integration into school routines [15]. These shortcomings highlight the need for participatory approaches that move beyond top-down designs. A promising strategy to address these challenges is group concept mapping, which offers a structured approach to integrating the perspectives of children, teachers, and stakeholders, thereby aligning more closely with real-world needs and strengthening its potential for long-term effectiveness and transferability [16].

Group concept mapping is a structured participatory methodology that systematically collects, integrates, and visually represents stakeholders’ perspectives [17]. By combing qualitative and quantitative methods [18,19,20,21,22,23], it enables the identification and prioritization of features that influence the usability of and engagement with health interventions [24]. Previous research supports that applying concept mapping in youth contexts [25] can reveal children’s genuine motivators (e.g., playful route expansions, scoreboard rivalry, cross-curricular quizzes) while also clarifying teacher concerns (e.g., limited time, alignment with academic schedules).

Given the increasing integration of smartphones and tablets into children’s daily routines, and the growing use of mobile applications for health promotion, it is essential to identify the features that make these tools engaging, accessible, and useful. Group concept mapping provides a structured way to achieve this by systematically incorporating the perspectives of primary school students, teachers, physical activity stakeholders, and families.

Within the Walk around the Earth (E-Walk) project [26,27], this participatory approach will first aim to identify the most influential factors affecting engagement with and usability of a mobile application designed to support physical activity in primary school students. Based on these insights, the project will then develop and refine a step-based app integrating educational and physical activity content, and subsequently evaluate its impact on (i) physical activity metrics (e.g., daily steps, minutes of activity), (ii) anthropometric parameters such as Body Mass Index (BMI), and (iii) changes in physical activity classification. Overall, the project seeks to address one of today’s most pressing health challenges by developing an intervention that combines physical activity, digital technologies, and learning opportunities, thus making physical activity more engaging and enjoyable.

## 2. Materials and Methods

### 2.1. Ethical Approval and Experimental Approach to the Problem

The E-Walk project obtained ethical approval from the Institutional Review Board of the Department of Human Sciences, Society and Health at the University of Cassino and Lazio Meridionale (Approval No.: 19764, dated 16 July 2025). To establish a comprehensive European framework of physical inactivity among children aged 9 to 15 years, the project will adopt an ethnographic approach integrated with concept mapping. This design will allow the exploration of children’s relationships with mobile apps, together with the perceptions of both students and teachers from diverse countries and contexts. By combining real-world insights with scientific expertise, this study aligns with an obligation–opportunity conceptual model.

Given the relatively broad age range (9 to 15 years), potential differences in motivations, developmental stages, and physical activity behaviors will be acknowledged and accounted for in the analyses by including age as a covariate or stratifying results by age group. The study primarily focuses on the design and development stages through group concept mapping, with the subsequent evaluation phase planned as a quasi-experimental pre-post study to assess the app’s impact on physical activity and sedentary behaviors.

### 2.2. Participants and Inclusion/Exclusion Criteria

Participants will include primary school students aged 9 to 15, schoolteachers, physical activity stakeholders, and relatives or guardians. Students will be subsequently divided into two age groups (9 to 12 and 13 to 15). Inclusion will be restricted to those regularly enrolled in primary schools and able to read and understand the language of the validated questionnaires used in each country. Only children with access to a compatible mobile device, either personal or shared, will be admitted. Those with medical conditions or physical disabilities that contraindicate moderate-to-vigorous physical activity, or with cognitive or developmental impairments that prevent them from comprehending the app content or questionnaire items, will not be eligible.

Teachers will be eligible if employed at one of the participating schools or directly involved in school activities. They must be able to complete the questionnaires and engage with app-related tasks, possessing sufficient digital literacy to use a smartphone or tablet. Adults with severe health conditions preventing participation in physical activity or those unable to engage with digital tools will be excluded.

Parents, guardians, and other stakeholders will be included if directly involved in supporting children’s education or physical activity and available to participate in focus groups, surveys, or feedback activities related to the intervention. Those lacking sufficient access to digital devices and literacy to engage with the app-based activities will be excluded.

Recruitment will take place through workshops organized in one or more local schools, ensuring a diverse range of ages and socio-cultural backgrounds. Participation will be voluntary, and all participants will be fully informed about the project and its aims. They will be allowed to withdraw from the study at any time without consequences. Written informed consent will be obtained from the participants. For children, written parental consent will also be required. In accordance with Regulation (EU) 2016/679 of the European Parliament and Council on the protection of personal data (General Data Protection Regulation), each participant will be assigned an anonymous identification code. All collected data will be used exclusively for statistical and research purposes.

### 2.3. Project Design and Implementation

Institutions interested in participating in the project’s activities will be involved. The E-Walk project will be carried out in several sequential phases, aimed at identifying the key features/factors/characteristics influencing children’s engagement in physical activity through the use of a mobile application. Data collection will occur in two main phases. In Phase 1 (Section 2.4), group concept mapping will be used to identify key features and inform the design of the app and its in-app challenges. Phase 2 (Section 2.5) will assess the impact of the deployed app and challenges, with data collected at baseline and follow-up. Baseline measures will include questionnaires and anthropometric data, process measures will track app usage and challenge completion, and outcome measures will assess changes in physical activity, sedentary behavior, and BMI.

### 2.4. Phase 1: Development (Concept Mapping and App Design)

**Figure 1:**
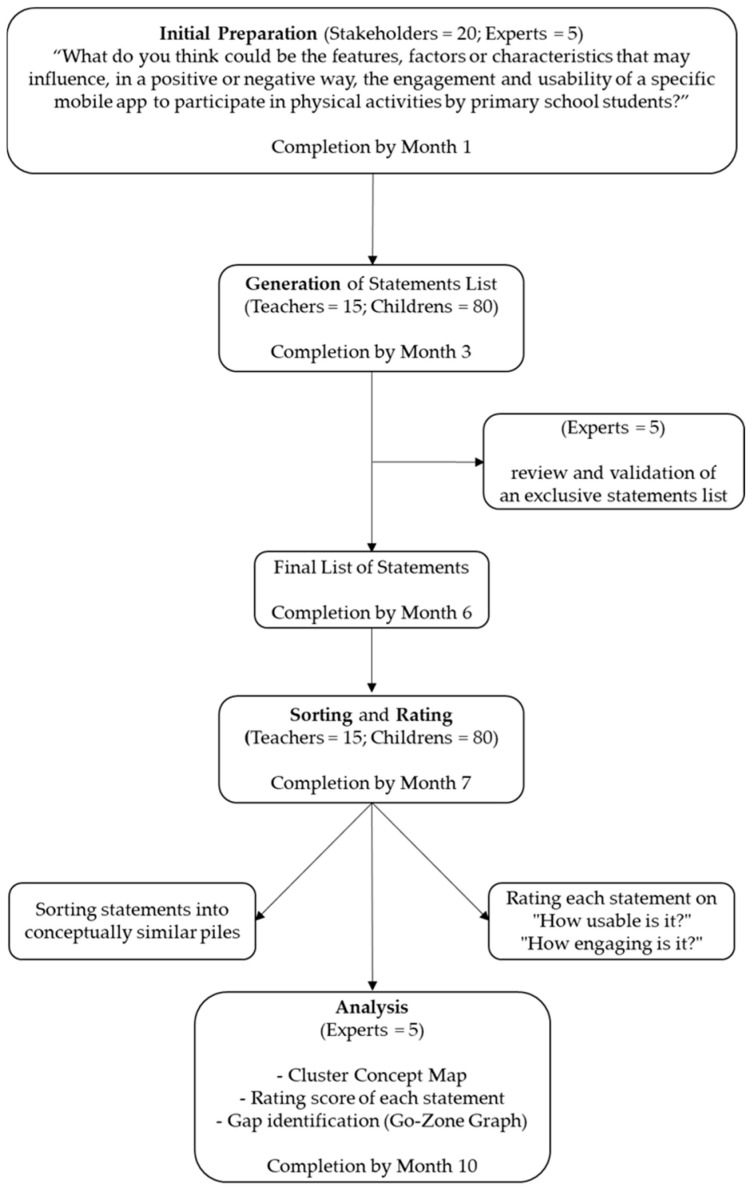
Flow diagram of the group concept mapping process.

Standardized operating protocols will be implemented by the E-Walk team to ensure consistency in data collection. The concept mapping process will include four main phases to provide a wide range of perspectives and to strengthen the conceptual framework, as shown in Figure 1 [24]:

1. Initial preparation: definition of the focus prompt.
2. Generation, review, and validation of an exclusive feature/factor/characteristic list (minimum 45, maximum 130 exclusive features/factors/characteristics).
3. Structural organization (sorting and rating): Stakeholders sort and rate features/factors/characteristics.
4. Data analysis and interpretation: The final phase will involve both a quantitative and qualitative analysis of the generated sorting and rating data. Specifically, the analysis will synthesize the viewpoints of children and teachers into a conceptual framework reflecting the influence of different features/factors/characteristics that may positively or negatively affect engagement with and usability of a mobile application designed to support physical activity participation in primary school students.

#### 2.4.1. Initial Preparation

In this phase, the focus prompt will be finalized to facilitate participant recruitment and stimulate idea generation during brainstorming activities. The proposed prompt will be

“What do you think could be the features, factors or characteristics that may influence, in a positive or negative way, the engagement and usability of a specific mobile app to participate in physical activities by primary school students?”

#### 2.4.2. Generation, Review, and Validation of an Exclusive Statement List

A comprehensive list of features/factors/characteristics will be gathered from brainstorming sessions involving children and teachers. These will be organized into a structured list of relevant features/factors/characteristics, representing potential influences on engagement with and usability of the mobile app. Online brainstorming sessions will be distributed across the eight European Union countries involved in the project, using networks of schools and professional associations.

To analyze the open-ended responses of the brainstorming phase, three researchers will create an initial codebook based on the focus prompt answers. Subsequently, two researchers will independently code each participant’s response, refining the codebook as necessary. This review will aim to ensure clarity, avoid repetitions, and decide whether any of the identified features/factors/characteristics need to be further broken down. Expert researchers with a deep knowledge of the subject area and a good command of English will be invited to evaluate and provide insights on the features/factors/characteristics. Each feature/factor/characteristic will be evaluated using a 5-point Likert scale, ranging from 1 (unclear) to 5 (clear), to assess its clarity. Researchers will have the opportunity to comment on the relevancy, representativeness, ratability, and saturation of each feature/factor/characteristic. This feedback will help to refine and enhance the quality of the list. In accordance with established guidelines, each feature/factor/characteristic that receives an evaluation score below 3 will be revised to improve clarity or entirely removed from the list [28]. Discrepancies will be discussed collaboratively until a consensus is achieved with the supervision of a third researcher. After completing the processes, a comprehensive and exhaustive list will be uploaded to Concept Systems software (Concept Systems, Incorporated, NY, USA, version: build 2022.273.21) for the subsequent structuring phase (sorting and ratings).

#### 2.4.3. Structural Organization (Sorting and Rating)

The structuring phase will comprise two distinct activities: sorting and rating. Upon providing participant information and consent, participants will be presented with the final list of features/factors/characteristics in a random sequence via the Concept Systems online platform.

Initially, the features/factors/characteristics will be presented in a random order, and the participants will use the Concept Systems software to categorize the features/factors/characteristics identified in the generation phase by sorting them based on perceived similarities. Each feature/factor/characteristic will be labeled with a unique code to indicate its origin, whether from the teachers, students, or other stakeholders. Participants will also be provided with an identification code to guarantee a consistent uniform interpretation. Each feature/factor/characteristic will be included in only one category/pile, and participants will be encouraged to sort the factors into a minimum of 6 to maximum of 14 categories/piles. Each category will be named to reflect its theme or content. Each feature/factor/characteristic will be assigned to only one category, ensuring that no feature/factor/characteristic will belong to multiple categories. If a feature/factor/characteristic appears distinct or unique, it could stand alone within its own category.

The rating phase will be conducted via an online survey in which participants will rate each feature/factor/characteristic on two six-point Likert scales: usability (1 = not usable, 6 = very usable) and level of engagement (1 = not engaging, 6 = very engaging).

#### 2.4.4. Data Analysis and Interpretation

Before starting the data analysis, one of the co-authors with certified expertise in the concept mapping procedure will review the participants’ sorting and rating responses to confirm their adherence to the provided guidelines. Descriptive statistics including mean, standard deviation, and frequency of occurrence will be calculated for both demographic data and feature/factor/characteristic rankings. Subsequently, the GroupWisdom™ online platform will be used for data analysis, employing a square symmetric similarity matrix generated from the sorted features/factors/characteristics. A two-dimensional non-metric multidimensional scaling technique will be applied, mapping each feature/factor/characteristic as a point on an x-y spatial “point map” for each cohort. Hierarchical cluster analysis will be utilized to cluster the points representing each feature/factor/characteristic on the point maps. For the children, teachers, and different stakeholders involved, the E-Walk project team will assess various cluster solutions following the recommended steps by Kane and Trochim [29] with the objective of collapsing clusters logically to emphasize distinct themes among the features/factors/characteristics. This process represents a mixed-method approach to the data analysis, blending the quantitative statistical outcomes with a qualitative interpretation by the project team. Then, clusters will be named based on category names provided by participants during the sorting phase and the consensus of the project team. Go-Zones will be established once the cluster maps are agreed upon and finalized, showcasing feature/factor/characteristic ratings on usability and level of engagement in relation to mean ratings. The top-right quadrant (green—IV) of the Go-Zone will comprise features/factors/characteristics considered both more important and more engaging than the average. Features/factors/characteristics and clusters identified in the concept map will be considered as candidate items (and groups) for inclusion in future reporting guidelines for concept mapping research [30].

### 2.5. Phase 2: Evaluation (Deployment and Impact Assessment)

The concept mapping procedure will be used to identify features/factors/characteristics that make a mobile app easy, enjoyable, and useful for encouraging physical activity among children based on the perspectives of primary school students, schoolteachers, physical activity stakeholders, and relatives and guardians [17]. In addition to informing the app’s design and usability features, the concept mapping approach will also guide the development of educational content as well as content related to physical activity and sport, ensuring that it will be aligned with the needs, preferences, and motivational drivers of these target groups. Once the mobile app and the sport activities (i.e., routes, tasks, challenges) are developed, students and other stakeholders will walk, run, hike, or roll along selected routes while engaging with educational and sport content derived from the concept mapping results. Feedback and user characteristics will be assessed as detailed in Section 2.5.1 and Section 2.5.2.

In line with the study’s objectives, specific success indicators will be used to evaluate the impact of the intervention. Primary indicators will include changes in physical activity metrics such as daily step count, minutes of moderate-to-vigorous activity, and reductions in sedentary time, as recorded through app-based measures and validated questionnaires. Physical activity levels will be assessed using the Physical Activity Questionnaire for Older Children (PAQ-C) [31] for children (students aged 9–15 years) and the short version of the International Physical Activity Questionnaire (IPAQ) [32] for teachers (adults aged ≥ 18 years old). The PAQ-C is a self-administered tool for children, providing a score from 1 (low activity) to 5 (high activity) based on nine items. The tenth item, related to illness, is not included in the final score. The PAQ-C has been validated in different countries and cultural contexts, demonstrating good reliability for assessing self-reported physical activity among school-aged children [31]. It has been translated into multiple languages and adapted to local habits (e.g., types of common sports or leisure-time activities), which makes it suitable for use in our multinational study context. The IPAQ includes 7 items assessing frequency, intensity, and duration of physical activity, along with sitting time. It includes both categorical (inactive, minimally active, HEPA active) and continuous scores (MET-minutes/week) [33,34]. The IPAQ has been validated for adults in more than 20 countries, with standardized translations and cultural adaptations [32]. In addition, anthropometric parameters will be collected as complementary success indicators to monitor changes in body composition and overall health status during the project. Weight and height will be determined using a precision instrument that combines a scale and stadiometer accurate to 0.1 kg and 0.1 cm (Seca, model 709, Vogel & Halke, Hamburg, Germany). Additionally, the BMI will be calculated. Both physical activity levels and anthropometric data will be evaluated for the entire three-year duration of the project.

#### 2.5.1. Activity Metrics and Educational Content

The activity metrics and data to be collected through the mobile app will be defined by the results of the group concept mapping conducted in the initial phases of the project. In line with the general objectives of the E-Walk project, and to comprehensively monitor both physical and educational engagement, the collected data will be organized into three main categories:

- Anthropometric Data: related to children’s growth and body composition, defined according to health promotion priorities.
- Physical Activity Parameters: measuring level, type, and intensity of activity, determined by technical feasibility and user preferences, and PAQ-C.
- Activity Enjoyment and User Feedback: To evaluate participants’ perceptions regarding the proposed activities, including their motivational impact, enjoyment, and usability. The tools and methods used to collect this type of feedback will be tailored according to the results of the concept mapping process, the app’s development, and the preferences identified among children and other stakeholders.

Educational content will also be integrated in the app to stimulate cognitive engagement. Its structure and topics will be developed based on concept mapping results, ensuring alignment with user needs and supporting the overall project objectives.

#### 2.5.2. Planned Mobile App Architecture and Functionalities

To address transparency regarding the technical and functional design of the E-Walk app, a dedicated description of the app’s planned architecture is provided below. While the app is still under development, the design roadmap and anticipated features are outlined to ensure clarity and reproducibility. The E-Walk application will be developed as a cross-platform native app using interface development technologies that ensure compatibility with both Android and iOS devices. Data storage and backend functionalities will rely on a platform providing secure, cloud-based management of activity metrics, user profiles, and educational content. This architecture enables real-time data synchronization, offline use, and scalable data handling. The key functionalities will be as follows:

- Physical activity tracking: Integration of GPS tracking and step counters to record activity duration, distance, and intensity. Data will be complemented by user-reported activity logs.
- Educational modules: short interactive quizzes and mini-games designed to stimulate cognitive engagement, aligned with topics identified through concept mapping.
- User interaction modes: options for individual challenges and team-based competitions, allowing flexible engagement and promoting social motivation.
- Feedback collection: in-app surveys, ratings, and motivational prompts to capture user enjoyment, preferences, and suggestions.
- Gamification elements: points, badges, and leaderboards to reinforce engagement and reward consistent participation.

All collected data will be anonymized and securely stored on the Firebase platform, with strict adherence to data protection regulations. Parents and teachers will have access to summarized reports of physical activity and educational engagement.

The app will follow an iterative development process:

1. Prototype phase: initial interface and feature mockups tested with a small sample of children and teachers.
2. Pilot testing: functional beta version deployed to evaluate usability, data collection accuracy, and engagement.
3. Full deployment: iterative refinement based on pilot feedback, including scaling educational content and activity tracking features.

This structured design ensures that the E-Walk mobile application will be robust, user-friendly, and aligned with the needs and expectations of primary school students.

### 2.6. Statistical Analysis

The evaluation phase of the project will adopt a quasi-experimental pre-post design without randomization. Children and schools will first participate in the baseline assessment (anthropometric data, PAQ-C/IPAQ, app usage, and engagement metrics), followed by the implementation of the mobile app and sport activities. Post-intervention assessments will be conducted at multiple time points (e.g., 6 months and 12 months after implementation) to examine changes in physical activity levels, sedentary behaviors, and anthropometric outcomes relative to baseline. The statistical methods and analyses that will be applied to investigate the effect of the mobile app, specifically designed based on a concept mapping approach, on physical activity engagement and educational interaction in primary school students are specifically detailed in Supplementary File S1 [35,36].

All analyses will be performed using STATA 18 (StataCorp LP, College Station, TX, USA).

## 3. Discussion

The E-Walk project seeks to propose a different approach to promoting physical activity and cognitive engagement in school-aged children through the development of a mobile application informed by community-based participatory research, such as group concept mapping. Given the increasing concern about sedentary behavior and excessive screen media use among youth, particularly following the COVID-19 pandemic, the E-Walk intervention is designed to address a key public health priority by exploring strategies to counteract inactivity while integrating digital tools with educational opportunities.

Within the E-Walk project, a step-based mobile app will be developed and subsequently evaluated, integrating both educational and physical activity content and aligning with the needs, preferences, and motivational drivers of its young users. Following its development, the project’s secondary aim is to examine whether the application succeeds in increasing physical activity levels and reducing sedentary behaviors among participating children and stakeholders.

Recent studies have shown the potential of mobile health apps to increase physical activity levels in children and adolescents. A systematic review indicated that apps featuring behavioral strategies such as goal setting, feedback, gamification, and parental involvement can be effective in promoting healthy behaviors [37]. These strategies have been found particularly beneficial for preventing obesity and supporting regular physical activity. Similarly, a meta-analysis highlighted that smartphone-based interventions can enhance overall physical activity and increase step counts among youth populations [38]. However, despite these findings, key challenges remain. User engagement and app usability remain critical factors in determining the overall success of such interventions. Barriers such as non-intuitive user interfaces, limited personalization, and lack of social interaction features may reduce the effectiveness of mobile apps, especially among younger users. The present study is designed to address these challenges by incorporating stakeholder perspectives, particularly those of children and teachers, into app design and refinement. While many existing fitness and health apps for youth follow a top-down model [39], the E-Walk project employs group concept mapping to capture end-user insights and ideas, with the aim of increasing the likelihood of creating a relevant, engaging, and sustainable tool.

Group concept mapping has emerged as a valuable participatory research method in public health. It combines qualitative and quantitative techniques to produce visual representations of complex ideas, offering a comprehensive understanding of stakeholder perspectives [40]. This methodology has been successfully used in clinical quality improvement efforts, ensuring that interventions are adapted to the specific needs of the target population [41]. By co-creating interventions with participants, researchers may improve both the relevance and uptake of health promotion strategies. Unlike traditional top-down approaches, where interventions are designed and implemented by experts with limited input from the target population, co-creation actively involves participants in the development process. This has the potential not only to ensure that strategies are culturally and contextually relevant but also to foster a sense of ownership, which could significantly improve engagement and long-term adoption.

For children, incorporating features such as scoreboards, fun routes, and short quizzes might represent strategies worth exploring to turn physical activity into a game-like experience. These features could help keep children motivated and focused by offering immediate feedback, visible progress, and clear goals. For teachers, the app is intended to be evaluated as a potentially easy-to-use tool that may be integrated into the school day without requiring additional instructional planning. Furthermore, the integration of educational content within the mobile application will allow the project to investigate whether it can support both physical and cognitive development simultaneously. By tracking not only physical activity but also indicators of cognitive engagement and knowledge acquisition, the E-Walk project will explore the potential for a dual benefit: promoting physical activity and enhancing learning through interactive and enjoyable experiences. This approach aligns with contemporary educational needs, which emphasize integrating health promotion with digital literacy. Additionally, the project aims to generate insights that may contribute to broader policy frameworks such as the Sustainable Development Goals (SDGs), particularly SDG 3 (Good Health and Well-Being) and SDG 4 (Quality Education) [42], by advancing child health and supporting inclusive, technology-based education.

Family engagement is another essential component of the E-Walk project. Family involvement in behavioral interventions has been shown to significantly enhance physical activity outcomes in children. A systematic review of family-based interventions using smartphone applications demonstrated that parental support positively influences children’s physical activity and health behaviors [43]. Families can play a central role by facilitating access to digital tools, reinforcing routines, and encouraging shared participation in app-based challenges. By promoting a supportive home environment, the E-Walk project will examine the extent to which family involvement may further enhance children’s motivation and adherence to physical activity. Demographic differences among users, such as gender and academic year, should also be considered during both the design and analysis phases. Prior research has shown that mobile app usage tends to be higher among females and older students, suggesting that engagement may vary across groups [44]. These findings reinforce the importance of personalizing health-related applications to ensure accessibility and effectiveness across different user groups.

In summary, this protocol outlines a stakeholder-informed strategy for developing and evaluating a mobile application aimed at promoting physical activity and educational engagement in primary school students. By integrating user feedback through concept mapping, educational content, and context-specific features, the project seeks to create a child-centered digital solution to address sedentary behavior. The planned evaluation will provide evidence on the feasibility, usability, and potential effectiveness, thereby contributing to the growing field of digital health research focused on sustainable behavioral change in school-aged children.

## 4. Conclusions

This protocol outlines the rationale and methodological framework of the E-Walk project, which integrates group concept mapping with the development of a mobile app to promote physical activity and reduce sedentary behaviors. By engaging key stakeholders, children, teachers, families, and physical activity professionals, the project aims to ensure that the app is user-centered, contextually relevant, and sustainable. The evaluation phase, based on predefined success indicators, will generate evidence on the app’s effectiveness in improving physical activity levels, reducing sedentary time, and supporting healthier growth trajectories. The E-Walk intervention has the potential to contribute to school-based health promotion strategies, strengthen the integration of digital tools in education, and advance public health goals related to child well-being.

## 5. Limitations

This research design has some limitations that should be considered when interpreting its aims and potential outcomes. The reliance on self-report questionnaires (PAQ-C and IPAQ), while validated and widely used, may be affected by recall errors and social desirability bias, and participant recruitment could be subject to selection bias, as schools and families already interested in health promotion may be more likely to participate. The heterogeneity of the target age group (9–15 years) represents an additional challenge, since developmental differences can influence both the usability of the mobile app and children’s engagement. The project also depends on technological availability, which raises the risk of a digital divide, while variability in school resources and teachers’ workload may affect feasibility and consistency across contexts. Moreover, as highlighted by the literature [12] where digital health tools produced neutral or limited effects, maintaining user motivation and ensuring long-term sustainability are recognized difficulties. To mitigate these issues, the E-Walk project adopts a participatory group concept mapping approach to align the intervention with children’s, teachers’, and families’ perspectives, integrates objective indicators such as anthropometric and app-based engagement metrics to complement self-reports, and embeds educational and gamified elements to enhance adherence over time. These strategies are expected to reduce contextual barriers and increase the effectiveness and sustainability of the intervention compared with similar initiatives.

## Data Availability

No new data were created or analyzed in this study.

## Supplementary Materials

The following supporting information can be downloaded at: https://www.mdpi.com/article/10.3390/ijerph22091460/s1, File S1: Detailed statistical analysis.

## Abbreviations

The following abbreviations are used in this manuscript:

E-Walk	Walk around the Earth
PARQ-C	Physical Activity Questionnaire for Older Children
IPAQ	International Physical Activity Questionnaire
BMI	Body Mass Index
SDGs	Sustainable Development Goals
