# Supplementary material for: Designing, Developing, and Evaluating a Stakeholder-Informed Mobile App to Promote Physical Activity in Children
Source: Int J Environ Res Public Health. 2025 Sep 20;22(9):1460. doi: 10.3390/ijerph22091460 (PMC12469936; doi:10.3390/ijerph22091460)
Supplement: Supplementary file 1 [file ijerph-22-01460-s001.zip › ijerph-3845236-supplementary.pdf]

2.6.1. Descriptive Statistics

Descriptive statistics will be used to provide an overview of the sample characteristics and to summarize the dependent and independent variables. This includes calculating measures such as means, standard deviation, medians, ranges, and percentages for the relevant variables. These will describe key aspects of app usage, step count, time spent physically active, educational engagement (e.g., quiz scores), and participant characteristics (e.g., age, school level). For example, we will provide the following:

The mean and standard deviation of steps per activity (e.g., mean = 8.000 steps, standard deviation = 1.200 steps);

Percentage of children achieving high physical activity level;

Median and range for duration to complete the tasks to capture the central tendency and variability in these activity-related parameters (e.g., median = 45 minutes, range = 30-60 minutes);

2.6.2. Normality Testing

Normality of the data will be assessed to determine the statistical methods to be applied. For example:

The Shapiro–Wilk test will be applied to completion time data during the different tasks to assess their normality (Shapiro–Wilk p-value < 0.05, indicating non-normal distribution).

2.6.3. Correlation Analysis

To explore relationships between physical activity metrics and educational outcomes, Pearson's or Spearman's correlations will be applied, depending on variable distribution. Assuming a moderate effect size (e.g., $r = 0.4$) and a significance level of 0.05, a sample size of approximately 100 participants might be necessary. A power of 0.80 can be achieved with 100 participants. Sample size calculations will be performed using G*Power software (version 3.1.0) [35]. For example:

Pearson's correlation between educational content (i.e. quiz scores, independent variable) and number of steps per activity (dependent variable) (e.g., $r = 0.50$, $p < 0.001$).

Spearman's rank correlation between educational content (i.e. quiz scores, independent variable) and activity duration (dependent variable) (e.g., $\varrho = 0.05$, $p = 0.4$).

2.6.4. Multilevel Mixed-Effect Generalized Linear Models

To explore differences in app engagement, physical activity metrics, and educational outcomes across different levels of influence (e.g., individual, class, school, country), multilevel mixed-effect generalized linear models will be employed. The multilevel structure will make it possible to examine how these factors influence the likelihood of achieving different tasks (e.g., meeting daily step goals, completing educational quizzes) while accounting for the nested nature of the data. This analysis could provide information about how gender, physical activity level, country and application usage time interact to affect the different activity metrics. The sample size needed for multilevel mixed-effect models is influenced by the complexity of the analysis and the anticipated effect sizes. Assuming moderate effects and accounting for the nested and repeated data structure, a minimum sample of approximately 300 individuals may be necessary. Under these conditions, a statistical power of 0.80 is expected to be attainable.

2.6.5. Generalized Estimating Equation Regression Models

Participants' baseline characteristics, physical activity level, step features (e.g., length, number), and activity performance (e.g., speed, total time), quiz scores will be modeled using generalized estimating equation regression models. For example, a strong positive association between quiz scores and activity performance could be identified, indicating that participants who performed better in the educational tasks also tended to complete the physical activities more efficiently, potentially reflecting the impact of the mobile application during physical activity in children. This approach accounts the correlated nature of the data, where multiple measurements will be evaluated from each participant across different activities, making it suitable for assessing the interaction between different variables and their influence on physical activity engagement. Assuming moderate effects, a sample of 200 participants should be sufficient to attain a statistical power of 0.80.

2.6.6. Nonlinear Regression Models

Nonlinear regression models may be applied to investigate complex relationships. The required sample size for such models will depend on the complexity of the regression equation and the effect sizes. Assuming moderate effects, a minimum of approximately 120 data points will be necessary to ensure sufficient statistical power (≥ 0.80). For example:

A nonlinear regression model may be applied to examine the relationship between physical activity performance (e.g., total distance covered, dependent variable) and predictors such as application usage time and physical activity level (e.g., distance = 2.5 − 0.3 × usage time + 4 × physical activity level − 0.1 × usage time × physical activity level).

2.6.7. Confounding Variable Management

The statistical analysis includes a comprehensive set of analyses aimed at ensuring the robustness and validity of the study findings. Across the different models applied, the sample size is expected to range from 100 to 300 participants, depending on the complexity of each analysis. To minimize the influence of potential confounders and to control for external sources of variability, participants will be assessed at baseline for factors that may affect physical activity engagement or educational outcomes. These may include prior physical activity habits, digital literacy, access to outdoor spaces, socioeconomic background, and school infrastructure. Detailed information will be collected on these potential confounders and will be incorporated as covariates in relevant statistical models. In particular, age-related heterogeneity will be explicitly addressed according to the literature by including age as a covariate in the models and, where appropriate, by conducting subgroup analyses (e.g. 9–12 vs. 13–15 years). This approach will allow us to explore whether differences in motivation, behavior, and app engagement are attributable to developmental stages within the 9 to 15 years range.

2.6.8. Inter-rater Agreement Analysis

To assess the consistency between raters evaluating the clarity of determinants using the Likert scales, inter-rater agreement will be calculated. Specifically, the weighted Cohen's kappa statistic will be used to account for the ordinal nature of the Likert scales, assigning greater penalty to larger disagreements. The kappa values will be interpreted according to Landis and Koch's benchmarks (e.g., 0.61–0.80: substantial agreement; 0.81–1.00: almost

perfect agreement). In cases involving more than two raters, Fleiss' kappa or intraclass correlation coefficients (ICCs) will be employed depending on the number of items and the rating structure. A kappa or ICC value ≥ 0.70 will be considered acceptable [36].

2.6.9. Missing Data Management

Missing data will be addressed under the assumption of data missing at random. Multiple imputation by chained equations (MICE) will be employed to impute missing values for both baseline and follow-up measures. Sensitivity analyses will be performed using complete-case analyses to assess the robustness of findings against different assumptions regarding missingness.

2.6.10. Analysis population

The primary analyses will follow the Intention-to-Treat (ITT) principle, including all participants with baseline assessment regardless of their level of adherence to the intervention. To evaluate the potential impact of adherence, complementary Per-Protocol (PP) analyses will be conducted, excluding participants with less than 50% engagement with the mobile application or participation in sport activities.

Supplementary Table S1. Mapping of research questions, hypotheses, and corresponding statistical analyses. This table summarizes the alignment between the main study objectives and the statistical methods selected for their evaluation.

Research Question (RQ)	Hypothesis	Primary Statistical Method
**RQ1.** Does the mobile application and associated sport activities increase physical activity levels (PAQ-C, steps, sedentary time) from baseline to follow-up?	Children using the mobile application will show higher PAQ-C scores, increased steps, and reduced sedentary time post-intervention compared with baseline.	Mixed-effect linear regression models for repeated measures, accounting for clustering at the class and school levels.
**RQ2.** Is educational engagement (quiz scores) associated with physical activity outcomes (steps, activity duration)?	Higher quiz scores will be positively associated with greater steps and shorter task completion times.	Correlation analysis (Pearson's or Spearman's depending on variable distribution).
**RQ3.** Do participant characteristics (e.g., gender, baseline activity) moderate intervention effects?	Intervention effects on physical activity will differ by gender and baseline activity level.	Interaction terms within mixed-effect regression models.
